# Global Telemedicine Implementation and Integration Within Health Systems to Fight the COVID-19 Pandemic: A Call to Action

**DOI:** 10.2196/18810

**Published:** 2020-04-02

**Authors:** Robin Ohannessian, Tu Anh Duong, Anna Odone

**Affiliations:** 1 Télémédecine 360 TLM360 Paris France; 2 Dermatology Department AP-HP, Henri Mondor Hospital Créteil France; 3 Chaire Avenir Sante numérique équipe 8 IMRB Inserm Créteil France; 4 School of Public Health Faculty of Medicine University Vita-Salute San Raffaele Milan Italy; 5 Clinical Epidemiology and HTA IRCCS San Raffaele Scientific Institute Milan Italy; 6 Digital Health Section European Public Health Association Milan Italy

**Keywords:** telemedicine, telehealth, digital health, digital medicine, COVID-19, coronavirus, SARS-CoV-2, public health, surveillance, outbreak, pandemic

## Abstract

On March 11, 2020, the World Health Organization declared the coronavirus disease 2019 (COVID-19) outbreak as a pandemic, with over 720,000 cases reported in more than 203 countries as of 31 March. The response strategy included early diagnosis, patient isolation, symptomatic monitoring of contacts as well as suspected and confirmed cases, and public health quarantine. In this context, telemedicine, particularly video consultations, has been promoted and scaled up to reduce the risk of transmission, especially in the United Kingdom and the United States of America. Based on a literature review, the first conceptual framework for telemedicine implementation during outbreaks was published in 2015. An updated framework for telemedicine in the COVID-19 pandemic has been defined. This framework could be applied at a large scale to improve the national public health response. Most countries, however, lack a regulatory framework to authorize, integrate, and reimburse telemedicine services, including in emergency and outbreak situations. In this context, Italy does not include telemedicine in the essential levels of care granted to all citizens within the National Health Service, while France authorized, reimbursed, and actively promoted the use of telemedicine. Several challenges remain for the global use and integration of telemedicine into the public health response to COVID-19 and future outbreaks. All stakeholders are encouraged to address the challenges and collaborate to promote the safe and evidence-based use of telemedicine during the current pandemic and future outbreaks. For countries without integrated telemedicine in their national health care system, the COVID-19 pandemic is a call to adopt the necessary regulatory frameworks for supporting wide adoption of telemedicine.

On March 11, 2020, the World Health Organization declared the coronavirus disease 2019 (COVID-19) outbreak as a pandemic, with over 720,000 cases reported in more than 203 countries as of 31 March. This announcement followed the declaration of a Public Health Emergency of International Concern (PHEIC) on January 30. The response strategy included early diagnosis, patient isolation, symptomatic monitoring of contacts, as well as suspected and confirmed cases, and a public health quarantine. The confinement of population and the outbreak impact on health care systems is disrupting routine care for non COVID-19 patients. In this context, telemedicine, particularly video consultations, has been promoted and scaled up to reduce the risk of transmission, especially in the United Kingdom [1] and the United States of America [2,3]. Telemental health services have been reported in China [4] and Australia as well [5].

Telemedicine was shown to be helpful in previous outbreaks, including former coronavirus outbreaks such as SARS-CoV (severe acute respiratory syndrome–associated coronavirus) and MERS-CoV (Middle East respiratory syndrome coronavirus), or PHEICs related to Ebola and Zika viruses [6,7]. Based on a literature review, the first conceptual framework for telemedicine implementation during outbreaks was published in 2015 [7]. The framework included tele-expertise, remote patient monitoring of contact cases, and teleconsultation for triage and isolated cases.

An updated framework for telemedicine during the COVID-19 pandemic has been defined in Figure 1. This framework could be applied at a large scale to improve national public health response, and should be shaped on the basis of scientific evidence arising from implemented telemedicine activities.

**Figure 1:**
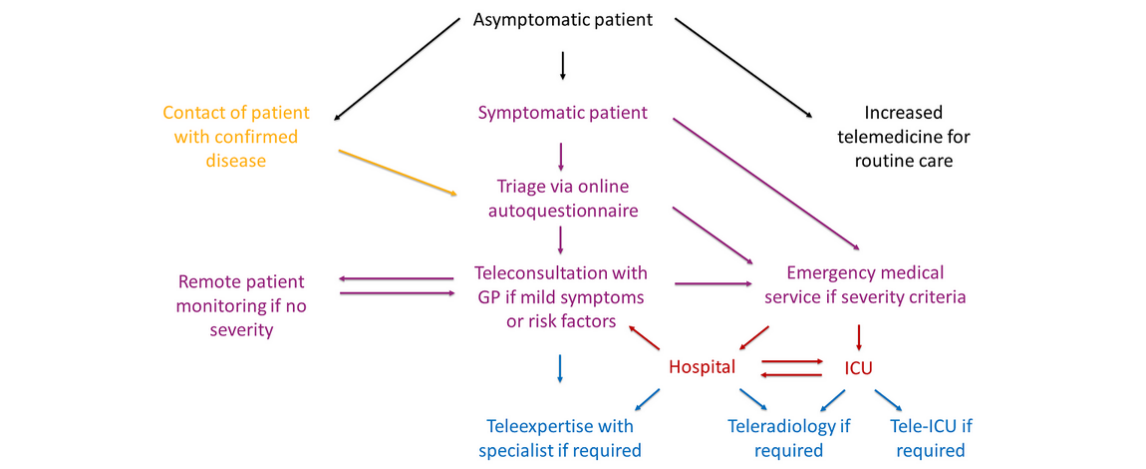
Conceptual framework of telemedicine for the coronavirus disease 2019 (COVID-19) pandemic. GP: general practitioner; ICU: intensive care unit.

Technological improvements and cost reduction of telemedicine solutions combined with both the high-speed internet and mass spread of smartphones makes it possible to apply this framework and quickly deploy video teleconsultations from a patient’s home.

Most countries, however, lack a regulatory framework to authorize, integrate, and reimburse telemedicine in their care delivery for all patients, particularly in emergency and outbreak situations [8]. Two possibilities are currently available for patients: (1) direct-to-consumer telemedicine with private providers mostly relying on out-of-pocket or private insurance payment and (2) free solutions, mainly from US-based companies (for example, WhatsApp, Skype, or Facetime), that may not respect national health data privacy and security requirements. Although these solutions may be useful to support and alleviate the pressure on health care systems during the outbreak, to date, they are mostly unintegrated within national health care systems and not sharing data with public health authorities for epidemiological surveillance.

With the second largest burden of COVID-19 in the world, Italy does not include telemedicine in the essential levels of care granted to all citizens within the National Health Service. No formal input was given on telemedicine by health authorities, despite high pressure on health services during the first phase of the epidemic [9,10]; not until an open call for telemedicine and monitoring system technologies proposals on March 24th was jointly issued by the Ministry for Technological Innovation and Digitalization, the Ministry of Health, the National Institute of Health and the WHO [11].

In France, the Ministry of Health signed a decree on March 9, 2020, allowing the reimbursement of video teleconsultations and tele-expertise by the National Health Insurance (NHI), for patients with COVID-19 symptoms and those confirmed with COVID-19 throughout the country, without the need to know the patient beforehand [12]. The decree was aimed to decrease unnecessary travel for medical consultations, limit the number of individuals grouping in waiting rooms, screen and detect suspected patients, and allow follow-up of mild confirmed cases from home. As the outbreak worsened, temporary funding for follow-up by nurses via video or phone as well as video teleconsultations by midwives (March 19, 2020) and speech therapists (March 25, 2020) was legally allowed.

The pre-existing telemedicine regulations also enabled primary care and hospital doctors to switch scheduled face-to-face consultations with known patients to reimbursed teleconsultations, when suitable. This model was activated in the largest national public academic hospital (AP-HP) in Paris, to encourage mass use of outpatient teleconsultations to reduce patient visits to the hospital (March 13, 2020). This has been reinforced by the High Council of Public Health, which recommended prioritization of teleconsultations for people with risk factors for severe disease in primary care (March 14, 2020) [13], followed by clinical and practical guidelines for patient examination by video consultation published by the Ministry of Health (March 16, 2020) [14]. Between 23 and 29 March, on the second week of national confinement, 486,369 teleconsultations were invoiced to the NHI, representing around 11% of all consults of the week [15]. Among general practitioners, 44% conducted at least one teleconsultation. Until early March, less than 10,000 teleconsultations a week were invoiced to the NHI.

In this context, several challenges remain for telemedicine to be globally used and integrated into the public health response to COVID-19 and future outbreaks:

1. The integration of telemedicine into international and national guidelines for public health preparedness (in keeping with International Health Regulations, 2005) and response [16]
2. The definition of national regulations and funding frameworks for telemedicine in the context of public health emergencies
3. A strategy to quickly define telemedicine frameworks; use case scenarios; develop clinical guidelines; and standardize triage auto questionnaire and remote patient-monitoring algorithms for any outbreaks at local, national, or global scales
4. A strategy and operational plan guiding health care providers to switch to outpatient teleconsultations and increase tele-expertise and remote patient monitoring
5. A communication toolkit to inform and educate the population on the recommended use of telemedicine
6. A data-sharing mechanism to integrate telemedicine providers’ data with epidemiological surveillance
7. A scientific evaluation framework and dedicated research funds to describe and assess the impact of telemedicine during outbreaks

All stakeholders are encouraged to address the challenges and collaborate to promote the safe and evidence-based use of telemedicine during the current pandemic and future outbreaks. For countries without integrated telemedicine within their national health care system, the COVID-19 pandemic is a call to adopt the necessary regulatory changes supporting wide adoption of telemedicine.

## Abbreviations

COVID-19: coronavirus disease 2019
MERS-CoV: Middle East respiratory syndrome coronavirus
PHEIC: Public Health Emergency of International Concern
SARS-CoV: severe acute respiratory syndrome–associated coronavirus
